# The Negative Impact of Triptolide on the Immune Function of Human Natural Killer Cells

**DOI:** 10.3390/ph16030458

**Published:** 2023-03-18

**Authors:** Na Wang, Xiaoyun Min, Ning Ma, Zhuoran Zhu, Bo Cao, Yuan Wang, Qing Yong, Jingjin Huang, Ke Li

**Affiliations:** 1 Core Research Laboratory, The Second Affiliated Hospital, Xi’an Jiaotong University, Xi’an 710004, China; 2 Department of Geriatric Digestive Surgery, The Second Affiliated Hospital, Xi’an Jiaotong University, Xi’an 710004, China; 3 Genertec Universal Xi’an Aero-Engine Hospital, Xi’an 710016, China

**Keywords:** TwHF, rheumatoid arthritis, CD107a, intracellular signaling, NK-activating receptor

## Abstract

Triptolide (TP), a bioactive compound extracted the from traditional Chinese medicine *Tripterygium wilfordii* Hook F (TwHF), has been shown to be effective in treating several autoimmune diseases, and has suppressive effects in several key immune cells such as dendritic cells, T cells, and macrophages. However, it is unknown whether TP has an impact on natural killer (NK) cells. Here, we report that TP has suppressive effects on human NK cell activity and effector functions. The suppressive effects were observed in human peripheral blood mononuclear cell cultures and purified NK cells from healthy donors, as well as in purified NK cells from patients with rheumatoid arthritis. TP treatment induced downregulation of NK-activating receptor (CD54, CD69) expression and IFN-gamma secretion, in a dose-dependent manner. When exposed to K562 target cells, TP treatment induced inhibition of surface expression of CD107a and IFN-gamma synthesis in NK cells. Furthermore, TP treatment induced activation of inhibitory signaling (SHIP, JNK) and inhibition of MAPK signaling (p38). Thus, our findings demonstrate a previously unknown role for TP in NK cell functional suppression and reveal several key intracellular signaling that can be regulated by TP. Our findings also offer new insight into mechanisms of TP therapeutic treatment in autoimmune disease.

## 1. Introduction

Triptolide (TP), the immunosuppressive compound derived from *Tripterygium wilfordii* Hook F (TwHF), has reportedly been successful in treating a number of autoimmune diseases, such as rheumatoid arthritis (RA) [1]. However, the mechanism of action of TP in autoimmune disease therapy remains elusive. Previous studies have shown that TP can suppress the production of pro-inflammatory cytokines and chemokines, [i.e., tumor necrosis factor-alpha (TNF-α), interleukin-2 (IL-2), IL-6, IL-8, and interferon (IFN)-γ], and regulate multiple immune cell functions [2]. T cell proliferation, activation, and immune regulation are all affected by TP [3]. It can also inhibit the IL-17 mRNA transcription and IL-6-triggered phosphorylation of signal transducers and activators of transcription 3, which are the crucial signaling molecule participating in Th17 cell development [3]. Research has shown that TP inhibits phenotypic changes and the maturation [4,5] and differentiation of dendritic cells (DCs) by suppressing CD1a, CD40, CD80, and CD86 expression while upregulating the expression of CD14 [3]. In addition, TP has been shown to impair the DCs’ capacity in eliciting allogeneic T cell responses [6], and downregulate the synthesis of C3, CD40, and B7h in renal tubular epithelial cells (non-classical antigen-presenting cell) were significantly downregulated in response to TP treatment [7,8]. In a murine model of adjuvant-induced arthritis (AA), TP reduced neutrophil recruitment, inhibited the pro-inflammatory cytokines’ expression status in neutrophils, and promoted neutrophil apoptosis [9]. TP can also induce the apoptosis of synoviocytes in RA [2]. Nevertheless, TP’s impact on NK cell functioning and the underlying mechanism has not been studied.

Natural killer (NK) cells are crucial for bridging the gap between innate and adaptive immunity. NK cells, as the innate immune system-related substantial effector cells, can destroy tumor or virus-infected cells without prior antigen exposure. Simultaneously, NK cells can regulate acquired immunity and are remarkably linked to the onset and progression of various immune diseases [10]. NK cells can sense different external stimuli and regulate their activity to play the corresponding immune function [11] by balancing signals received from inhibitory receptors and activating receptors on their surface. NK cells, for example, can spontaneously kill cells lacking self-antigen markers by secreting granulin and perforin, and regulate the functions of other important immune cells by secreting cytokines [12]. Evidence from recent research has indicated that abundant NK cells are present in inflammatory joints of patients with RA or other arthritic diseases [13], which play a pathogenic role in autoimmune diseases. Reportedly, NK cells isolated from the synovial fluid of patients with RA can induce the differentiation of CD14+ monocytes into osteoclasts [14,15]. Using anti-asialoGM1, the NK cell depletion in the model reduced both bone erosion and joint inflammation [14]. According to recent research, chemokine receptors (i.e., CCR1, CCR5, and CXCR3) are expressed by synovial NK cells, which can facilitate the recruitment of inflammatory cells (driven by their respective chemokines) into the RA synovium [16], prime effector myeloid cells, and aggravate arthritis by producing inflammatory mediators such as granulocyte-macrophage colony-stimulating factor (GM-CSF), macrophage (M)-CSF, and receptor activator of nuclear factor (NF)-kappaB ligand (RANKL) [17,18].

Scientific studies have shown that TP has negative regulatory effects on multiple immune cells such as dendritic cells, T cells and neutrophils [19]. However, it is unknow whether TP has significant effects on NK cells. Given the NK cells are an important player in various autoimmune diseases [14], determining the effects of TP on NK cells would improve our understanding of NK cell functional regulation and TP therapeutic mechanisms. In the present study, we investigated the role of TP in regulating human NK cell function and its relevance to RA.

## 2. Results

### 2.1. TP Did Not Influence the Proportion of Peripheral Blood NK Cells in Normal Human PBMCs

**Figure 1:**
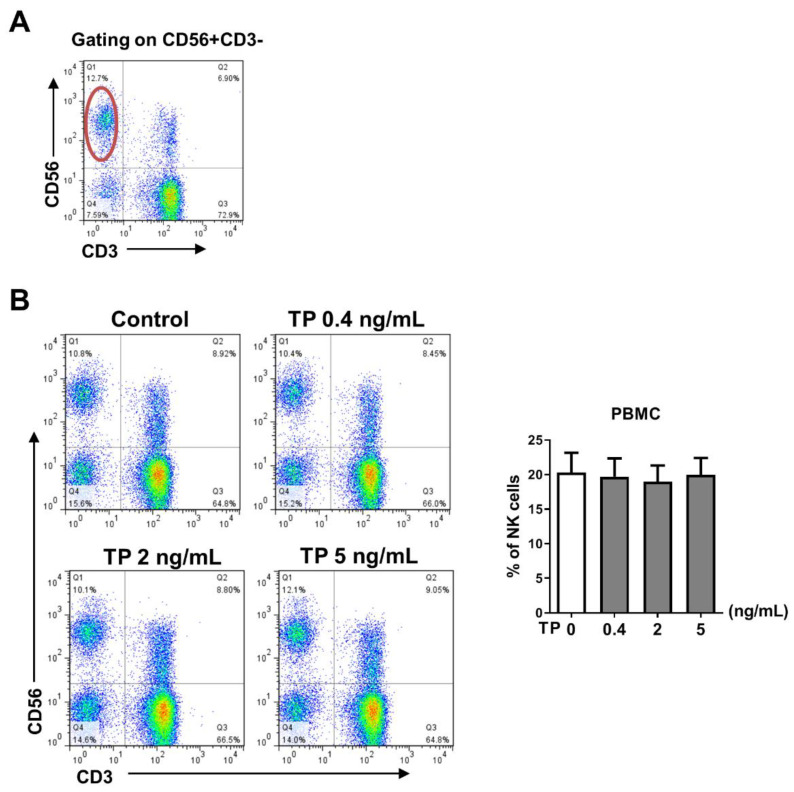
Effect of TP on the proportion and the viability of peripheral blood NK cells in normal human PBMCs. Human PBMCs isolated from health donors were incubated with different concentrations of TP (i.e., 0, 0.4, 2, and 5 ng/mL) for 24 h. (**A**) Representative FACS analysis shows the gating strategy of CD56+CD3−cells (NK cells). (**B**) The percentage of CD56+CD3−cells was detected by flow cytometry in PBMCs. Data were analyzed by one-way analysis of variance with a multiple comparisons test (*n* = 6).

In order to determine whether TP has an impact on the distribution of NK cells in normal human PBMCs, PBMCs were freshly isolated from peripheral blood and stimulated with TP in various concentrations (i.e., 0, 0.4, 2, and 5 ng/mL) for 24 h. The percentage of CD56+CD3− NK cells was measured in the TP-treated PBMCs (Figure 1A). Our data indicated that no significant difference in NK cell proportion was observed in PBMCs exposed to different concentrations of TP (Figure 1). 

### 2.2. TP Inhibited the Activity and Function of NK Cells in Normal Human PBMCs

**Figure 2:**
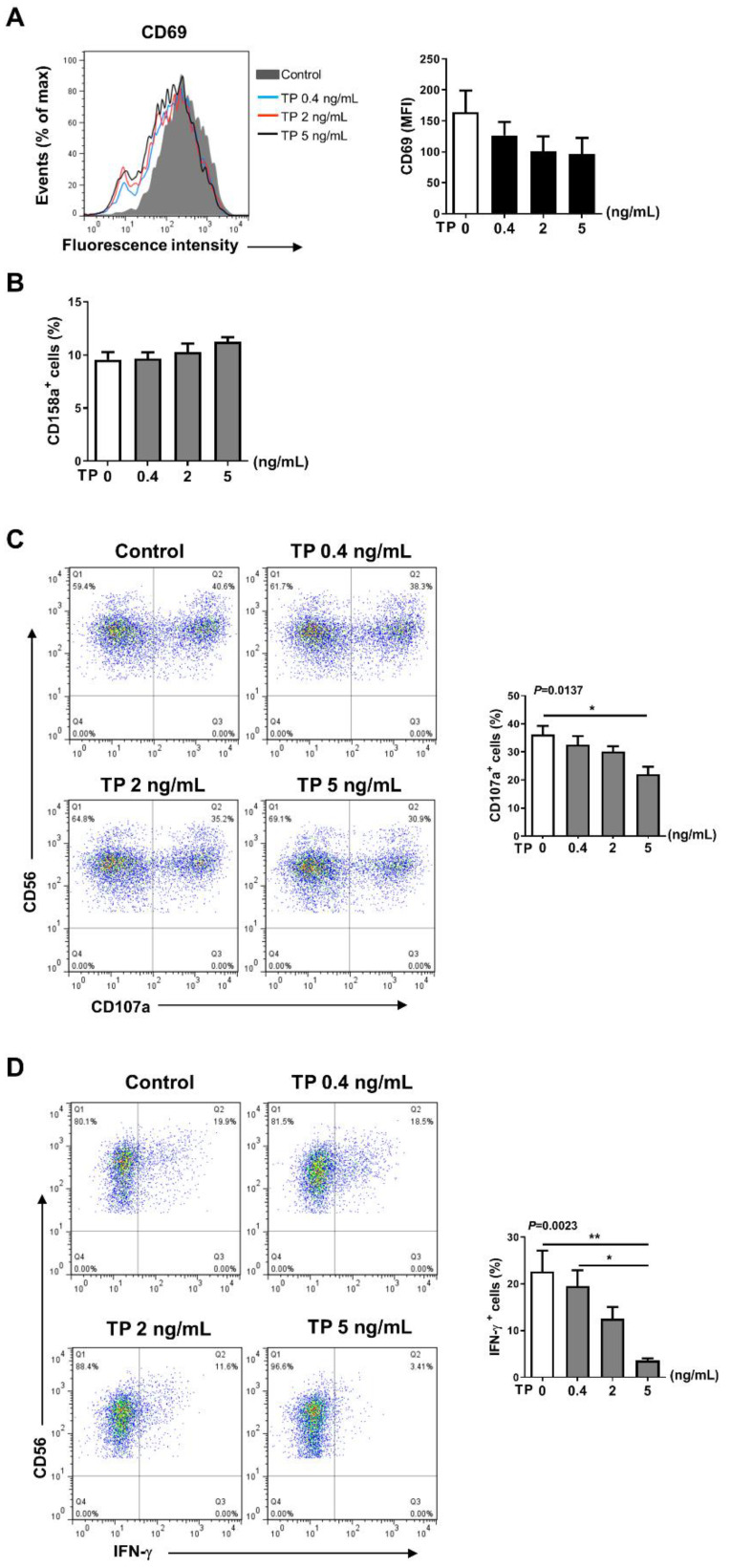
Effects of TP on the phenotype and function of NK cells in human PBMCs. Human PBMCs from health donors were treated with TP as shown (i.e., 0, 0.4, 2, and 5 ng/mL) for 24 h. (**A**,**B**) The expression of CD69 (**A**) and CD158a (**B**) were detected by flow cytometry in CD56+CD3−cells. (**C**) PBMCs were further co-cultured with K562 cells at a ratio of 2:1 (PBMC:K562 = 2:1), flow cytometry analysis of CD107a expression was carried out on CD56+CD3−cells. (**D**) The IFN-γ expression in CD56+CD3−cells was measured by intracellular staining and flow cytometry. Data were analyzed by one-way analysis of variance with a multiple comparisons test (*n* = 4), * *p* < 0.05, ** *p* < 0.01.

To assess the effects of TP on activity and subsequent immune reaction of NK cells, in vitro, freshly prepared human PBMCs were incubated with TP at various concentrations (i.e., 0, 0.4, 2, and 5 ng/mL) for 24 h. We first assessed surface phenotype activity of NK cells in PBMCs. The results of flow cytometry showed that expression of the activating receptors CD69 was downregulated in a concentration-dependent manner, whereas the inhibitory receptor CD158a became up-regulated on NK cells in PBMCs, in a TP concentration-dependent manner, compared with untreated cells (Figure 2A,B). Expression of the activating receptors NKp46, CD54, and the inhibitory receptor CD158b was not significantly altered during TP treatment (The following supporting information can be downloaded at: https://www.mdpi.com/article/10.3390/ph16030458/s1, Figure S1: Effects of TP on the phenotype of NK cells in human PBMCs). Accordingly, we found a marked reduction in the expression of CD107a, an NK cell functional marker, on NK cells in PBMCs by incubation with TP, following the stimulation with K562 tumor cells (Figure 2C). Likewise, we observed that IFN-γ expression was also significantly attenuated in TP-treated PBMCs, at 5 ng/mL concentration, which decreased IFN-γ expression by approximately fourfold (Figure 2D). Collectively, these findings suggest that TP negatively regulates NK cell activity and function in PBMCs.

### 2.3. TP Had Negative Effects on Human Purified NK Cell Activity and Function

**Figure 3:**
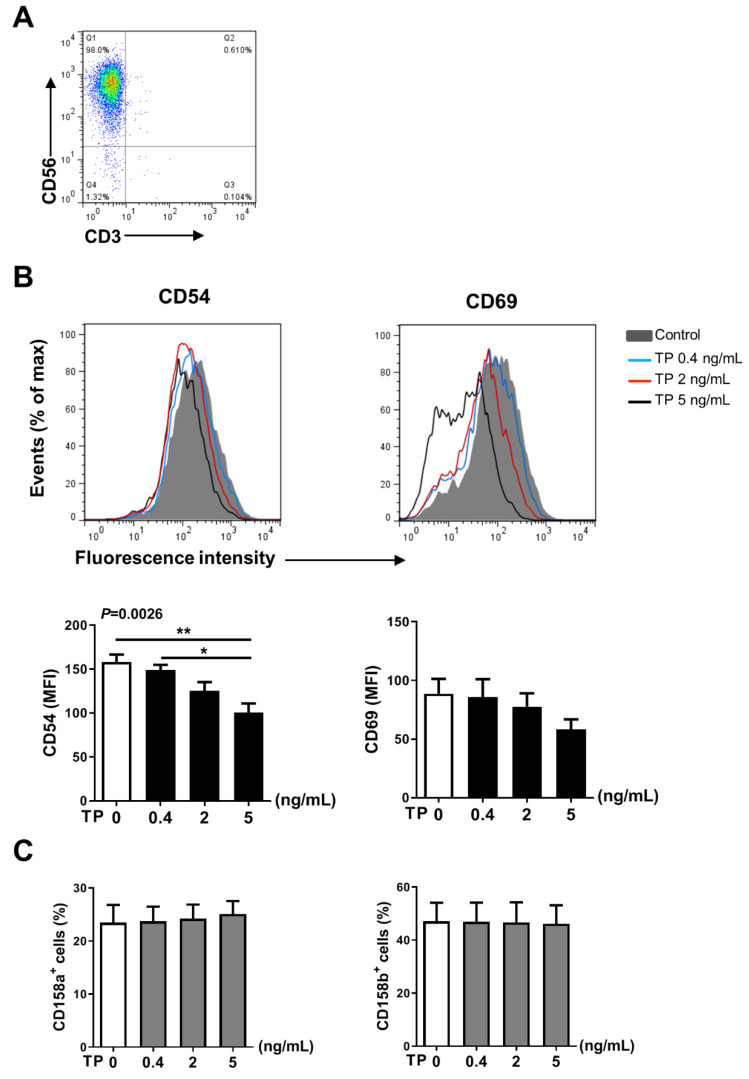
Effects of TP on the phenotype of human purified NK cell. (**A**) Human purified NK cells were isolated from human peripheral blood mononuclear cells by immunomagnetic negative sorting. (**B**,**C**) Purified NK cells were stimulated with TP as indicated (i.e., 0, 0.4, 2, and 5 ng/mL) for 24 h, the expression of the activating receptors CD54, CD69 (**B**) and the inhibitory receptors CD158a, CD158b (**C**) were measured by flow cytometry. Data were analyzed by one-way analysis of variance with a multiple comparisons test (*n* = 4), * *p* < 0.05, ** *p* < 0.01.

**Figure 4:**
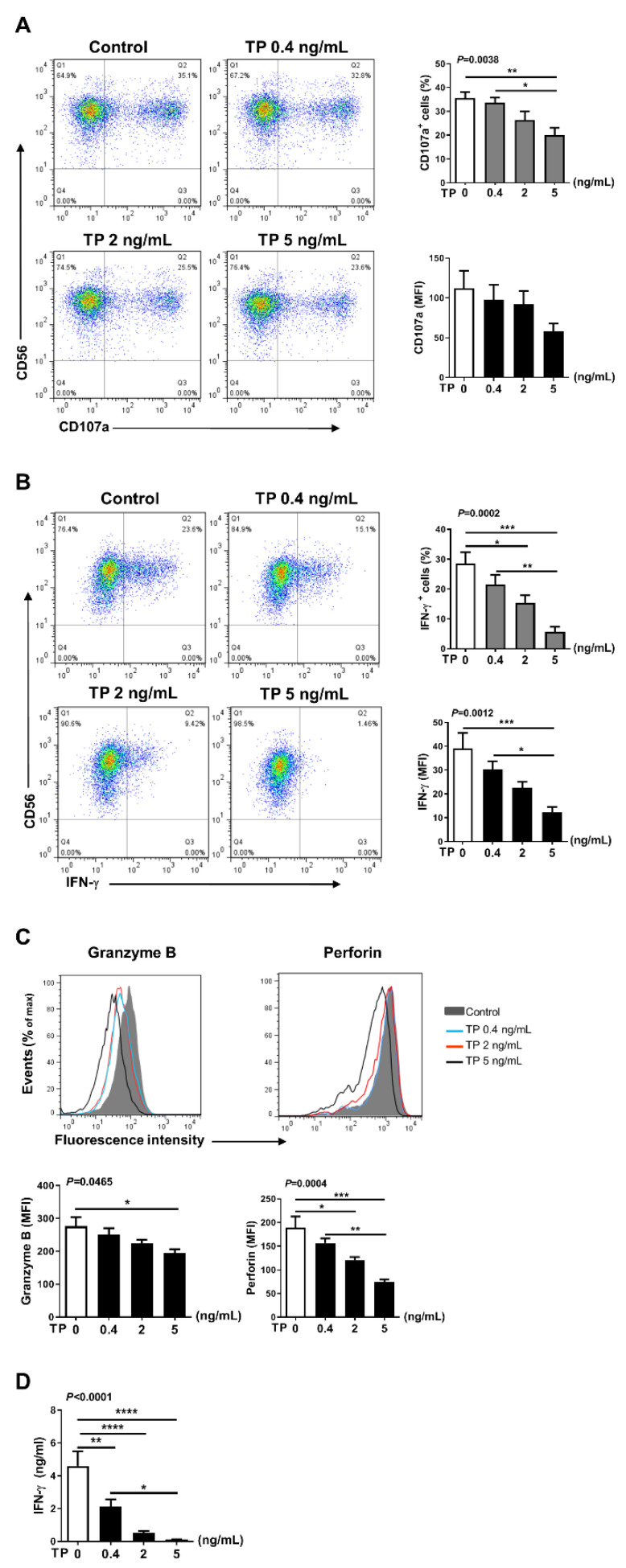
Effects of TP on the function of human purified NK cells. Purified human NK cells were treated with TP as indicated. Supernatants were collected after 24 h, and NK cells were further co-cultured with K562 at a ratio of 2:1 (NK:K562 = 2:1). (**A**,**B**) Flow cytometric analysis of CD107a was carried out on NK cells. The IFN-γ expression was tested by flow cytometry after cells were fixed and permeabilized. The results including percentage of positive cells and fluorescence intensity (MFI) are shown. (**C**) Granzyme B and Perforin expressions were measured by intracellular staining and flow cytometry. (**D**) The amounts of IFN-γ in supernatants was determined by ELISA. Data were analyzed by one-way analysis of variance with a multiple comparisons test (*n* = 4), * *p* < 0.05, ** *p* < 0.01, *** *p* < 0.001, **** *p* < 0.0001.

In addition to NK cells, there are many other immune cells in the peripheral blood PBMCs, the immune cells interact with each other. Therefore, in order to further verify the effects of TP on human NK cell activity and function, we isolated the NK cells from human PBMCs by immunomagnetic negative sorting (Figure 3A). After different concentrations of TP (i.e., 0.4, 2, and 5 ng/mL) treatment for 24 h, we examined the NK cell activity and function by flow cytometry. Our results showed that TP remarkably lowered the expression of activating receptors CD54 and CD69, but did not influence the levels of the inhibitory receptors CD158a and CD158b (Figure 3B,C). Similar to our PBMCs findings, following the co-incubation with K562 tumor cells, expression of NK cell functional markers (i.e., CD107a, IFN-γ, granzyme B, perforin) was significantly inhibited on the NK cells by stimulation with TP in a concentration-dependent manner (Figure 4A–C). Furthermore, we measured the IFN-γ level in supernatant of different groups of TP-treated purified NK cells by ELISA. As shown in Figure 4D, TP effectively decreased the production of this cytokine. Together, these results demonstrate that TP plays a crucial role in NK cell functional activity suppression.

### 2.4. Effects of TP on Intracellular Signalling in NK Cells

**Figure 5:**
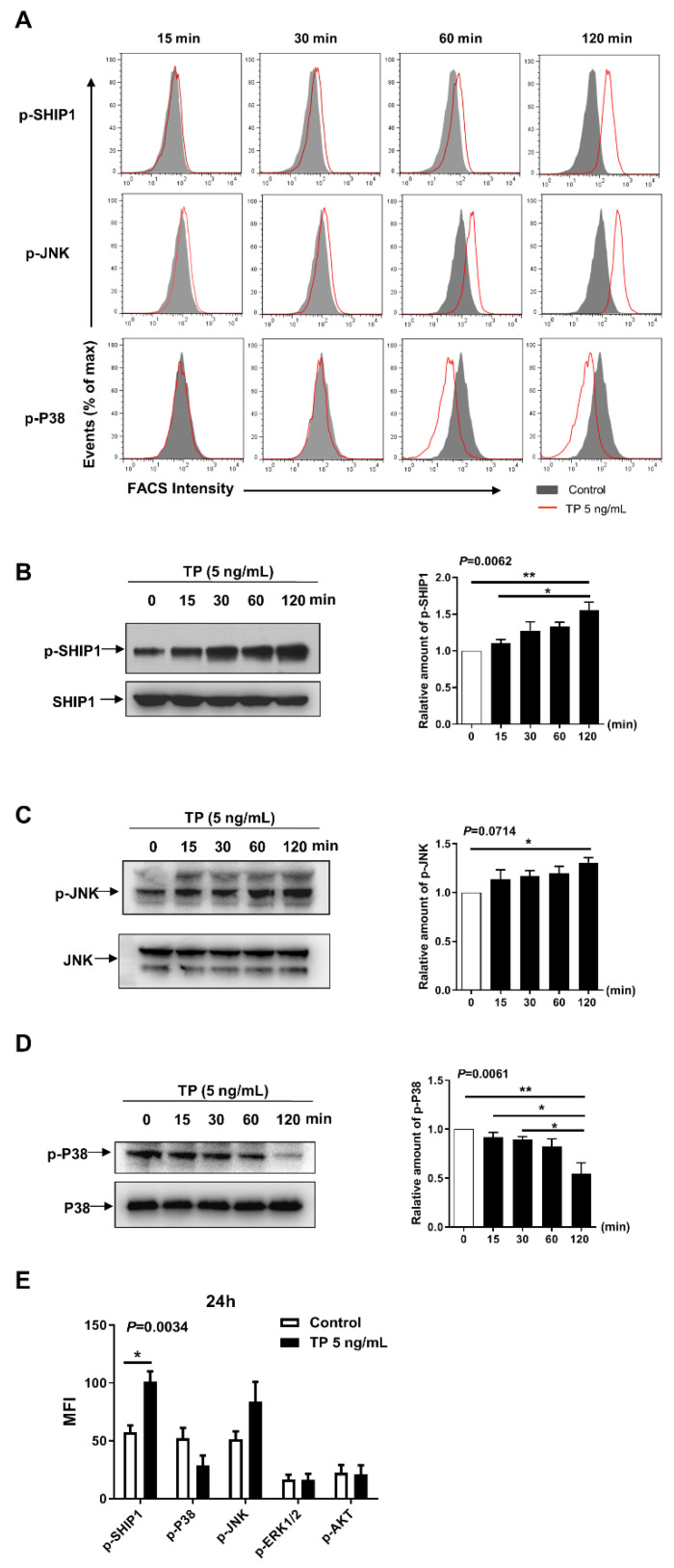
Effects of TP on intracellular signaling in NK cells. (**A**) Human NK cells were stimulated with TP (5 ng/mL) for 0/15/30/60/120 min, and then the expression of p-SHIP1, P-P38, and p-JNK were measured by flow cytometry. (**B**–**D**) Human NK cells were treated with TP (5 ng/mL) for indicated time periods. Cells were lysed and protein was extracted, the expression of p-SHIP1, p-P38 and p-JNK were tested by western blot. (**E**) Human NK cells were treated with TP (5 ng/mL) for 24 h, flow cytometric analysis of p-SHIP1, p-JNK, p-ERK1/2, p-P38 and p-Akt were carried out on NK cells. Data were analyzed by one-way analysis ((**B**–**D**), *n* = 3) and two-way analysis of variance with a multiple comparisons test ((**E**), *n* = 5), * *p* < 0.05, ** *p* < 0.01.

Previous studies have suggested that Src homology 2 (SH2) domain-containing inositol-5-phosphatase-1 (SHIP-1), c-Jun N-terminal kinase (JNK), and mitogen-activated protein kinase (MAPK) intracellular pathways have an important role in modulating the immunologic state and cellular interaction in many kind of cells, including NK cell [20]. To investigate the underlying molecular mechanisms of TP suppressing the activation and function of NK cells, we explored the effect of TP on the activation of SHIP-1, P38, JNK, ERK1/2, and AKT. NK cells were stimulated with TP for different times and the changes of phosphorylation of the above mentioned intracellular pathways were examined. We observed that TP treatment enhanced phosphorylation of SHIP-1 and JNK, but significantly suppressed the phosphorylated p38 in a time-dependent manner, as measured by flow cytometry (Figure 5A) and western blot (Figure 5B–D). There were no significant changes in the activity of ERK1/2 and AKT by TP stimulation (The following supporting information can be downloaded at: https://www.mdpi.com/article/10.3390/ph16030458/s1, Figure S2: TP stimulation does not affect ERK and AKT signalling in NK cells). In addition, the results of TP treatment for 24 h in NK cells were similar with TP stimulating for 2 h on the activation of signaling pathways (Figure 5E).

### 2.5. TP Impaired NK Cell Function of Rheumatoid Arthritis (RA) Patients without Treatment

**Figure 6:**
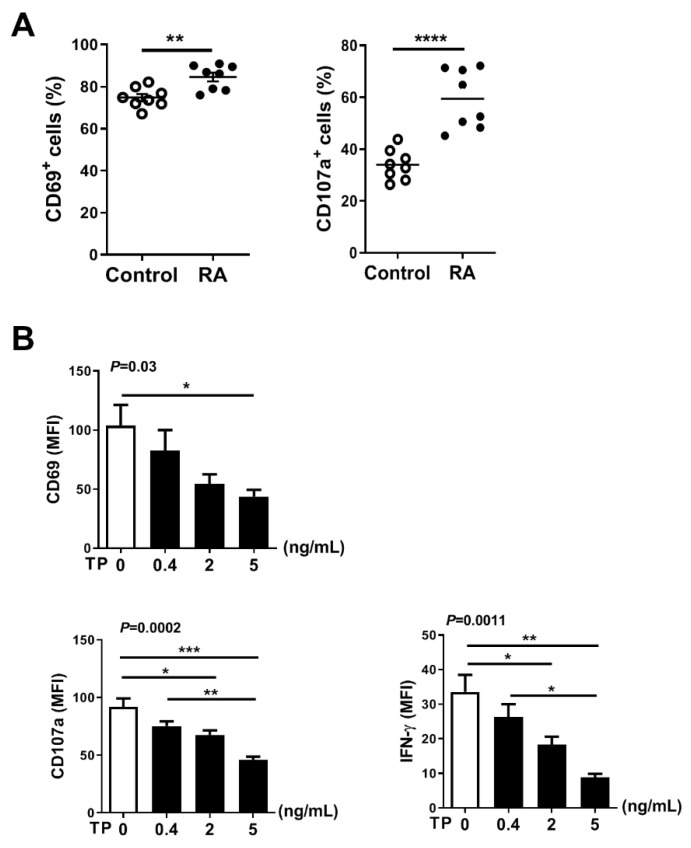
TP impairs NK cell function of rheumatoid arthritis patients without treatment. (**A**) From healthy donors and rheumatoid arthritis patients without any treatments, human PBMCs were isolated, respectively. The expression of CD69 was detected by flow cytometry in CD56+CD3−cells. PBMCs were further co-cultured with K562 cells at a ratio of 2:1 (PBMC:K562 = 2:1), flow cytometric analysis of CD107a expression was carried out on CD56+CD3−cells. (**B**) PBMCs from rheumatoid arthritis patients were treated with TP as indicated (i.e., 0, 0.4, 2, 5 ng/mL) for 24 h, the expression of CD69, CD107a and IFN-γ were measured by flow cytometry in CD56+CD3−cells. Data were analyzed by unpaired *t*-test ((**A**), *n* = 8) and one-way analysis of variance with a multiple comparisons test ((**B**), *n* = 4), * *p* < 0.05, ** *p* < 0.01, *** *p* < 0.001, **** *p* < 0.0001.

NK cell is thought to play a critical role in the occurrence and development of RA. To explore the activity and function of NK cells in RA patients, we first evaluated surface phenotype activity and functional markers of NK cells in PBMCs from RA patients (without received any treatment) and healthy donors. Flow cytometry analysis showed that the expression of activating receptors CD69 and function marker CD107a was significantly increased on NK cells from RA patients, compared with control healthy donors (Figure 6A). To further verify the role of TP in regulating the activity and function of RA NK cells, we co-cultured RA PBMCs with TP as indicated (i.e., 0, 0.4, 2, and 5 ng/mL) for 24 h and measured NK cell activity and function markers by flow cytometry. We found that RA NK cells that had been co-cultured with TP, especially at high concentration (5 ng/mL), exhibited significantly lower expression of activating receptor CD69 and function markers (CD107a, IFN-γ) than that control (untreated) cells (Figure 6B). Collectively, these findings demonstrated that TP is a potent negative regulator of RA NK cell activity and function. This conclusion might explain, in part, the therapeutic effect of TP on RA.

## 3. Discussion

TwHF has been used in folk medicine for hundreds of years as a treatment for various autoimmune diseases. However, the primary molecular mechanisms and cellular targets remain elusive. TP is a bioactive compound extracted from TwHF. Despite that the suppressive effects of TP have been documented in several key immune cells, it is unknown whether TP has an impact on NK cells. This study demonstrates that TP has a negative impact on human NK cell activity and function in vitro settings. The suppressive effect was observed in the NK cells from healthy donors as well as patients with RA.

NK cells have developed a tolerance for the self while differentiating into fully competent killers. The NK cells’ capacity of expressing a variety of activating receptors that bind to endogenous ligands can be attenuated through various rapidly evolving inhibitory receptor–ligand pairs. However, certain combinations of these pairs may be highly variable between individuals, the genetic polymorphism can provide a hypothetical possibility for NK cell-mediated auto-reactivity [21,22]. NK cell activation is accompanied by cytotoxic activity and pro-inflammatory cytokines secretion. These functions play important roles in immune surveillance. On the other hand, excessive NK functions could lead to clinical disorders such as autoimmune disease. It has been reported that in type I diabetes, NK cells can destroy pancreatic β cells in a manner dependent on NKp46 and promote islet destruction [23]. NK cells in the RA patients’ inflamed joints and psoriatic skin lesions may contribute to disease progression by increasing local inflammation [24,25]. Controlling NK cell induction and proliferation is therefore necessary to prevent them from developing into the contributor involved in autoimmune responses.

In this study, normal human PBMCs and purified NK cells were treated with TP at varying concentrations. Our data indicated that TP did not influence the proportion of peripheral blood NK cells in PBMCs (Figure 1B). However, TP inhibited NK cell activation and function in a concentration-dependent manner. This phenomenon was observed in both PBMC-NK cells and purified NK cells, with the effect being more significant in purified NK cells. Previous studies have demonstrated that, in addition to killing target cells by releasing cytotoxic granules (perforin and granzyme), activated NK cells can secrete pro-inflammatory cytokines. In this way, NK cells can induce inflammation, shape, and control other immune cells’ activities in the local microenvironment, and influence adaptive immune response formation by transmitting and amplifying these crucial cytokine signals, such as IFN-γ. Furthermore, TP was also observed to inhibit NK cell activation, killing function, and secretion of IFN-γ in PBMCs of patients with RA. These findings support the notion that TP-mediated NK cell suppression may contribute to the therapeutic effect of TP on autoimmune diseases such as RA.

In the present study, we identified several key intracellular signals which are important for NK cell activation/function that were regulated by TP. As a negative regulator of immune cells, SHIP (also known as SHIP1) has been reported to be phosphorylated after activating a variety of membrane receptors (e.g., B-cell receptor, Fc receptor, and T cell receptor) [20]. Many other intracellular signaling pathways (e.g., MAPK), can be modulated by SHIP via catalytic or non-catalytic activation [20]. Our results showed that TP stimulation induced an enhancement of SHIP-related phosphorylation, which was associated to a reduction of P38 phosphorylation. These observations are in line with earlier findings on the role of SHIP in inhibiting MAPK’s downstream signaling [26]. We observed that TP stimulation enhanced the JNK’s phosphorylation in NK cells, indicating that TP modulation of JNK is not dependent on SHIP. Therefore, our signaling study revealed the effects of TP on signaling transduction pathways; namely, activation of SHIP and JNK and suppression of P38.

There are limitations to this study. Our study was focused on determining the role of TP in NK cell activation/function regulation, and did not compare its effectiveness with those of recognized immunosuppressants such as cyclosporine A (CsA) in our in vitro model. In addition, our study did not address the effectiveness of TP on NK cell activation/function in vivo model (e.g., murine model of collagen-induced arthritis), which warrants further investigation.

## 4. Materials and Methods

### 4.1. Preparation of Human PBMCs and NK Cells 

From a panel of eight patients diagnosed with RA and ten healthy donors, human peripheral blood samples were obtained. Before the procedure, all the participants aged between 25 and 53 years provided informed consent. The Second Affiliated Hospital of Xi’an Jiaotong University’s Ethics Committee Board granted its approval for this research. By Ficoll-Hypaque gradient centrifugation method, the peripheral blood samples were mixed with PBS in equal volume, which was slowly added into the appropriate amount of lymphocyte separation solution (Pancoll human, P40-60500, Pan-Biotech/Pan-Seratech, Aidenbach, Germany), after density gradient centrifugation, the mixture was stratified, and PBMCs were obtained by gently drawing the white membranous cell layer in the middle with a Pasteur pipette. From PBMCs, the isolation of total NK cells was performed utilizing a NK Cell Isolation Kit (human) (130-092-657, Myltenyi Biotec, Bergisch Gladbach, Germany) following the guidelines of the manufacturer. In accordance with flow cytometry, the CD56+CD3- NK cell preparation’s purity after isolation was >90% routinely [27].

### 4.2. Cell Culture

Human PBMCs or purified NK cells (at a concentration of 10^6^/mL) were incubated in complete Rosewell Park Memorial Institute Medium-1640 (10% heat-inactivated fetal calf serum, 50 µg/mL streptomycin, 2 mM glutamine, 50 μM 2-mercaptoethanol and 50 U/mL penicillin) for 24 h containing 50 pg/mL recombinant human IL-2 (200-02-50UG, PeproTech, Cranbury, USA) in a humidified environment comprising 5% CO_2_ at a temperature of 37 °C [27,28]. In several trials, PBMCs or NK cells were cultured with a TP-containing medium for a predetermined period of time. TP (T3652, Sigma-Aldrich, Saint Louis, MO, USA) was dissolved in dimethyl sulfoxide, after reconstitution, stock solution was stored at −80 °C, avoiding repeated freeze–thaw cycles, and additional dilution was done with the culture medium to the indicated concentration during the experiments.

### 4.3. Human NK Cells Phenotypic and Functional Analyses

For NK cells phenotypic analysis, cultured PBMCs or purified NK cells were washed, and staining was performed for 30 min on ice utilizing a cocktail of directly conjugated antibodies against surface molecules, comprising APC anti-human CD56 (BioLegend, San Diego, CA, USA), FITC Mouse Anti-Human CD3 (BD Biosciences, San Diego, CA, USA), PE anti-human CD335 (NKp46), -CD54, -CD69, -CD158a, -CD158b (all are from BioLegend, San Diego, CA, USA). In some experiments that require analysis of NK cell function, cultured PBMCs or purified NK cells were washed and co-cultured with K562 cells at a 2:1 effector-target (E/T) ratio for 40 min. Fluorescent directly-labeled antibodies [i.e., anti-CD56 (APC), -CD3 (FITC), -CD107a (PE, BioLegend, San Diego, CA, USA)] were added and incubated on ice for 30min, then cells were further washed and fixed in a 2% formaldehyde solution, followed by flow cytometry analysis. For detection of granzyme B and perforin, the co-cultured cells were first performed membrane staining with anti-CD56 and anti-CD3 antibodies. After staining, the cells were fixed and permeabilised with a Fixation/Permeabilization Kit (BD Biosciences, San Diego, CA, USA) following the manufacturer’s instructions, intracellular staining was performed with fluorochrome-conjugated antibodies, PE Mouse Anti-Human Granzyme B (BD Biosciences, San Diego, CA, USA) or PE anti-human Perforin (BioLegend, San Diego, CA, USA), to investigate NK cell function under stimulation with target cells [20]. 

### 4.4. Interferon-γ (IFN-γ) Synthesis in NK Cells

To assess intracellular IFN-γ synthesis, cultured PBMCs or purified NK cells were co-cultured for 40 min with K562 cells before adding BD GolgiStop™ Protein Transport Inhibitor (comprises monensin, BD Biosciences, San Diego, CA, USA) and incubating for another 4 h [27]. Cells were used for detecting IFN-γ producing by intracellular staining. Supernatants of purified NK cells were used for measuring cytokine secretion by enzyme-linked immunosorbent assay (ELISA). PE Mouse Anti-Human IFN-γ antibody (BD Biosciences, San Diego, CA, USA) was then used to stain the cells intracellularly. Employing Fluorescence-Activated Cell Sorting Calibur (Becton Dickinson, San Jose, CA, USA), a flow cytometric examination was conducted. Utilizing FlowJo software (version 7.6.2, Tree Star, Ashland, OR, USA), data analysis was done. The positive cells’ percentage and mean fluorescence intensity were obtained. Employing Human IFN-γ ELISA Set (BD Biosciences, San Diego, CA, USA), IFN-γ concentrations in cell culture supernatants were obtained. According to the guidelines of manufacturers, the assays were performed.

### 4.5. Western Blot

After incubation with TP, NK cells were lysed at a predetermined period of time. Identical amounts of proteins were treated by sodium dodecyl sulfate-polyacrylamide gel electrophoresis and placed onto polyvinylidene difluoride membranes. An overnight incubation of membranes was performed at 4 °C with the primary antibody [i.e., anti-phospho-SHIP1 (Tyr1020), -phospho-SAPK/JNK (Thr183/Tyr185), -phospho-p38 MAPK (Thr180/Tyr182), -phospho-p44/42 MAPK (Erk1/2), -phospho-Akt (Ser473) antibodies, and anti-SHIP1 (D1163), -SAPK/JNK, -p38 MAPK, -p44/42 MAPK (Erk1/2), -Akt antibodies, all are from Cell Signaling Technology, Danvers, MA, USA], and then incubation with horseradish peroxidase-conjugated secondary antibody was conducted. Employing Amersham ECL Select™ detection reagent (GE Healthcare Life Sciences, Marlborough, MA, USA), visualization of protein bands was done. Protein bands on the gel were quantified by measuring the strength of individual bands using ImageJ software (version 1.47t, National Institutes of Health, Bethesda, MD, USA).

### 4.6. Statistical Analysis

Graphpad Prism software (version 8.0.1, LaJolla, CA, USA) was employed to perform the statistical analyses. Data were presented as mean ± standard error of the mean. Unpaired *t*-test was employed for the comparison between two groups with unmatched data. Multiple comparison test, either One-way or Two-way, was used to compare the means of >2 independent groups [29]. *p* < 0.05 indicated a statistical significance level. 

## 5. Conclusions

In summary, our findings demonstrate that TP has inhibitory effects on human NK cell activity/function in freshly isolated NK cells, in a TP dose-dependent (0–5 ng/mL) manner. Our findings also reveal that TP can activate SHIP-1 and JNK signaling and inhibit p38 signaling, which is in line with TP-mediated NK cell functional suppression. Furthermore, the finding that TP has inhibitory effects on RA patients’ NK cell activation/function provides new insight into mechanisms of TP therapeutic treatment in autoimmune disease.

## Data Availability

Data is contained within the article.

## Supplementary Materials

The followings are available online at https://www.mdpi.com/article/10.3390/ph16030458/s1, Figure S1. Effect of TP on the viability, phenotype and function of NK cells in normal human PBMCs. Figure S2. TP stimulation does not affect ERK and AKT signalling in NK cells.
