# Supplementary figures and images for: The Negative Impact of Triptolide on the Immune Function of Human Natural Killer Cells
Source: Pharmaceuticals (Basel). 2023 Mar 18;16(3):458. doi: 10.3390/ph16030458 (PMC10057343; doi:10.3390/ph16030458)

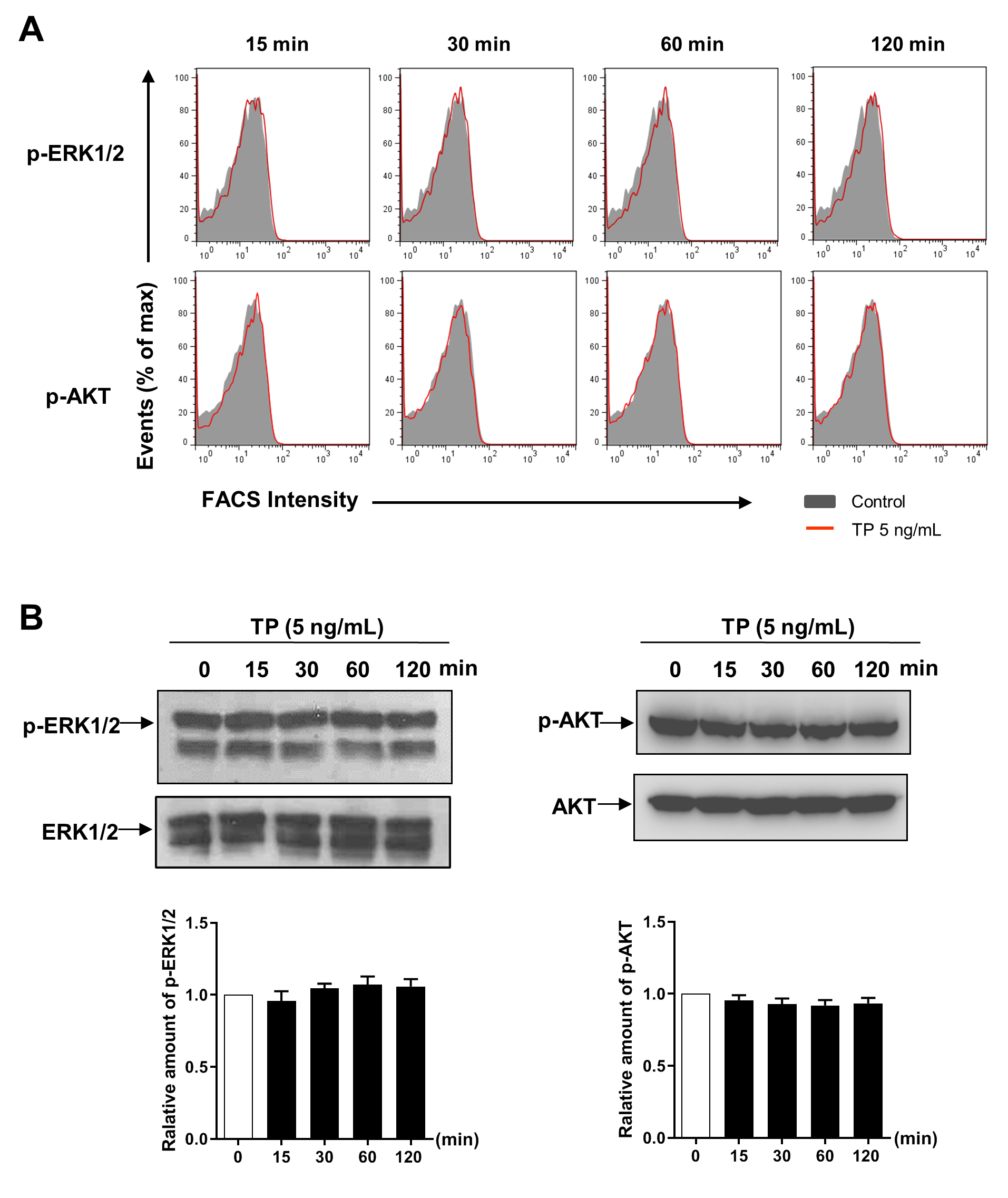

Supplement: Supplementary file 1 [file pharmaceuticals-16-00458-s001.zip › Figure S2 - high resolution.TIF]

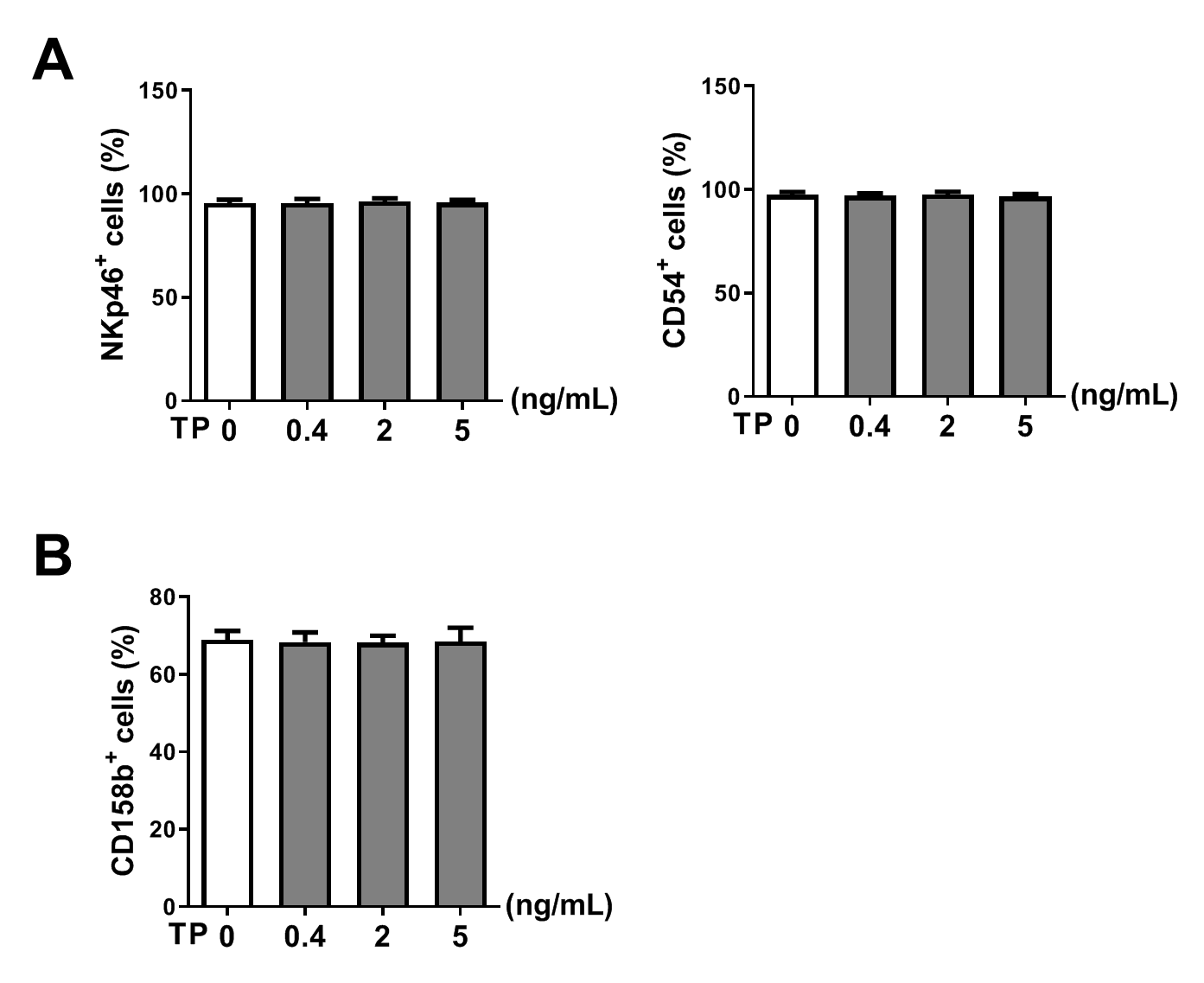

Supplement: Supplementary file 1 [file pharmaceuticals-16-00458-s001.zip › Figure S1 - high resolution.TIF]
